# Global, Regional, and National Disease Burden and Prediction Analysis of Colorectal Cancer Attributable to Tobacco, Alcohol, and Obesity From 1990 to 2030

**DOI:** 10.3389/fonc.2025.1524308

**Published:** 2025-02-26

**Authors:** Yuqi Deng, Yajie Wang, Jinsai Yang, Xinyu Luo, Jieya Qiu, Rou Long, Chaohui Zhang, Jiale Li, Guiyang Tang, Lili Chen, Jianhong Zuo

**Affiliations:** ^1^ Department of Health, Inspection and Quarantine, School of Public Health, University of South China, Hengyang, China; ^2^ Computer Institute, Hengyang Medical School, University of South China, Hunan, Hengyang, China; ^3^ The Affiliated Nanhua Hospital, Hengyang Medical School, University of South China, Hengyang, China; ^4^ Transformation Research Lab, Hengyang Medical School, University of South China, Hunan, Hengyang, China; ^5^ The Third Affiliated Hospital, Hengyang Medical School, University of South China, Hengyang, China

**Keywords:** burden of disease, colorectal cancer, smoking, alcohol use, high body mass index (high BMI)

## Abstract

**Background:**

Colorectal cancer (CRC) ranks among the highest in incidence and mortality rates globally. A significant portion of Colorectal cancer cases and deaths can be attributed to modifiable risk factors, with smoking, alcohol use, and high body mass index (BMI) being the three most prominent. However, the impact of these risk factors on Colorectal cancer across regions, genders, and age groups remains insufficiently characterized.

**Methods:**

Utilizing data from the Global Burden of Disease (GBD) study 2019, restrictive cubic splines (RCS) and quantile regression analyses are applied to explore the relationship between the Socio-Demographic Index (SDI) and ASMR or ASDR. Additionally, gender differences, changes across different SDI levels, and age group trends in smoking, alcohol use, and high BMI over the 30-year period are analyzed. The Bayesian age-period-cohort (BAPC) model is employed to predict mortality trends from 2020 to 2030, aiming to explore the epidemiological and sociodemographic transitions in the Colorectal cancer disease burden attributed to smoking, alcohol use, and high BMI.

**Results:**

In 2019, the number of colorectal cancer deaths globally attributable to risk factors as smoking, alcohol consumption, and obesity increased to 142,931, 52,495, and 85,882 cases respectively, collectively accounting for approximately one-third of all Colorectal cancer-related deaths. Notably, there is an upward trend in early-onset Colorectal cancer mortality associated with these factors.

**Discussion:**

To reduce the burden of Colorectal cancer, it is recommended to enhance health education, promote smoking cessation and alcohol moderation, and increase the coverage and participation in Colorectal cancer screening, which are crucial for lowering Colorectal cancer mortality rates. These findings are vital for the development of public health policies and intervention measures to reduce the global disease burden. They provide guidance for Colorectal cancer prevention across different regions, genders, and age groups worldwide.

## 1 Introduction

In the digestive system, colorectal cancer (CRC) is a prevalent cancer. Data from the 2019 Global Burden of Disease (GBD) study shows that CRC because of different risk factors has been the second most common cancer worldwide since 1990, ranking second among men after lung, tracheal, and bronchial cancers and second among women after cervical cancer; The years of life lost (YLL) are still rising, making the illness one of the top 10 causes of mortality globally (1). The disability-adjusted life years (DALYs) resulting from colorectal cancer rose by 96% between 1990 and 2019 (2), ranking it fourth among major illnesses and making a substantial contribution to the worldwide healthcare burden (3).

The incidence and mortality of colorectal cancer are primarily influenced by non-modifiable and modifiable risk factors (4). Modifiable risk factors are intimately linked to the majority of colorectal cancer cases and fatalities that result from the disease (5). Past studies indicate that modifiable risk factors for colorectal cancer include tobacco use, alcohol consumption, excessive fat intake, a history of diabetes, inflammatory bowel disease, and a high body mass index (6, 7); According to the current research analysis, the three major modifiable risk factors for colorectal cancer are smoking, alcohol consumption, and obesity (8). However, due to variations in risk factors among different countries and regions, as well as significant differences in economic development levels and population structures, the burden of colorectal cancer exhibits notable disparities (9, 10). According on geographic closeness and epidemiological similarities, the GBD 2019 divides all nations and regions into 21 areas (11); The incidence of colorectal cancer is substantially greater in industrialized regions like Australia, Europe, and North America than in developing regions like Asia, Africa, and Latin America, according current epidemiological statistics based on the GBD 2019 data. However, when compared to Asia and Africa, the rise in death rates in these industrialized regions is comparatively smaller (12, 13). This indicates that with the rise in economic levels, the incidence of colorectal cancer increases; however, improvements in healthcare quality have also led to a reduction in mortality rates. To achieve more precise and specific analysis, the GBD 2019 is divided into five categories based on the Socio-demographic Index (SDI): high (0.81-1), upper-middle (0.69-0.81), middle (0.61-0.69), lower-middle (0.45-0.61), and low (0-0.45) (14); This stratification method provides researchers and policymakers with an effective tool to compare health indicators and disease burdens across countries and regions with varying levels of development. Additionally, a standardized global framework is established to assess the disease burden and health outcomes across countries and regions (15, 16).Research data from the United States indicate that the overall incidence of colorectal cancer has decreased by nearly 40% as the coverage rate of colonoscopy screening in the 50 to 64 age group increased from 20% in 2000 to 61% in 2018 (17, 18), Effectively implementing population-based colorectal cancer screening programs lowers the rising disease burden. For specific situations, more precise colorectal cancer screening can be conducted.

To this end, this study collects and analyzes data on colorectal cancer attributed to smoking, alcohol use, and high body mass index across different SDI levels. The aim is to explore the mortality rate and the trends in age-standardized mortality rates (ASMR) of colorectal cancer caused by smoking, alcohol consumption, and obesity across varying SDI levels and regions. Additionally, a colorectal cancer risk prediction model is used to precisely intervene in high-risk populations across different age groups, with the goal of preventing and early detecting colorectal cancer.

From the research perspective, previous studies have mostly focused on individual risk factors of colorectal cancer, while we explored the juxtaposition of the three most important risk factors, and comprehensively considered the contribution of smoking, alcohol abuse, and high body mass index to the burden of disease of colorectal cancer from different regions, genders, and age groups. At the same time, we fill the gap between multifactorial comprehensive prevention and control and precision medicine, and provide new perspectives and a solid foundation for the development of colorectal cancer prevention and control policies in different regions of the world.

## 2 Methods

### 2.1 Research objective and data sources

Data relevant to colorectal cancer risk factors in this study are derived from the 2019 Global Burden of Disease (GBD) research. The GBD represents the largest and most comprehensive scientific study to date, aimed at quantifying health levels and their trends (19). The Institute for Health Metrics and Evaluation (IHME) at the University of Washington is leading the initiative, which is acknowledged as a genuinely worldwide study. The recent update is conducted by over 9,000 researchers from 162 countries and regions, providing data on premature deaths, diseases, disabilities caused by 370 conditions across 204 countries since 1990. These data are categorized by age and gender (20). In order to assess the prevalence of illnesses, injuries, and risk factors across different age groups, genders, nations, locations, and time periods, the GBD creates a special platform. Decision-makers, heads of health departments, researchers, and knowledgeable individuals can use this method to compare their nation’s health progress to that of other countries and to learn more about preventable causes that could result in serious health loss (21).

### 2.2 Mortality estimates

The GBD mortality database is generated through the GBD cause-of-death ensemble model using an algorithm known as CoDCorrect, which calculates the YLL by multiplying the CoDCorrect-adjusted estimate of the final death by the GBD standard life expectancy based on age, sex and place of death. Years of Disability Survival Estimates (YLD) for cancer are determined using weights for incidence, survival, and disability (22). The incidence rate is derived from the ratio of mortality to incidence, while the absolute survival rate is determined through the relative survival rate. The durations of the four post-cancer states (survival time less than 10 years) and two post-cancer states (survival time greater than 10 years) are combined with the corresponding disability weights to calculate YLD. To calculate Disability-Adjusted Life Years (DALYs), the Years of Life Lost (YLL and Years Lived with Disability (YLD for each age, gender, and location are summed. Age-standardized rates are calculated using the GBD 2019 global standard population data. The Population Attributable Fraction (PAF) is estimated using a comparative risk assessment framework that combines exposure levels and relative risks. The burden of a specific risk factor is calculated by multiplying the Population Attributable Fraction (PAF) by the corresponding burden of disease metric (23). The GBD cause of death ensemble model, combined with the CoDCorrect algorithm, provides a comprehensive framework for this study. It systematically assesses national trends in age-specific and gender-specific all-cause and cause-specific mortality rates. This approach helps quantify uncertainty estimates and enhances model reliability, while ensuring internal data consistency.

Based on similarities in disease spectrum, health risk factors, accessibility and quality of healthcare services, and geographic proximity, All nations and regions are divided into 21 categories. Furthermore, the Social Demographic Index (SDI), as a comprehensive indicator for measuring the level of social development in countries and regions, reflects a region’s population status and development level through an integrated assessment of various socioeconomic and demographic data (24). The calculation method of the SDI may draw from the Human Development Index (HDI). It combines per capita income, the average years of education for those aged 15 and above, and the total fertility rate for those under 25 to form a composite indicator, with values ranging from 0 to 1. According to the Social Demographic Index (SDI), countries and regions are categorized into five groups: high (0.81-1), upper-middle (0.69-0.81), middle (0.61-0.69), lower-middle (0.45-0.61), and low (0-0.45). This stratification method provides researchers and policymakers with a convenient means to compare health indicators and disease burdens across countries and regions with different levels of development.

### 2.3 Statistical analysis methods

#### 2.3.1 Restricted cubic spline

It is a non-parametric regression technique widely used in the field of data analysis and modeling. It is based on the principle of spline function, through the range of independent variable into multiple intervals, in each interval to build low order polynomial function (usually cubic polynomial), and ensure that the function connection point (i. e., node) at continuous and first, second derivative continuous, to flexibly approximate complex nonlinear function relationship. Its smoothness helps to more accurately describe the changes in survival risk over time or occurring together with other variables, thus improving the survival predictive power of the model. The advantage of the Restricted Cubic Splines (RCS) method lies in its ability to flexibly capture and model nonlinear relationships between survival time and continuous variables. Furthermore, In order to simulate survival curves, RCS converts multiple survival times into piecewise functions at different nodes. However, this segment construction function makes RCS to adapt to all variety of irregular, nonlinear data mode, can closely fit the actual trend of nonlinear data, capture the data hidden subtle trend, thus significantly improve the model of data fit, reduce the residual, unlike like simple linear model confined to the line fitting. Another advantage of RCS is that it can flexibly set the number and location of nodes according to the characteristics of the data, and customize the fitting for the nonlinear relationships with different degrees of complexity; smoothness facilitates a more accurate depiction of changes in survival risk over time or with other variables, thereby enhancing the model’s predictive capacity for survival. Depending on the sample size, 3 to 5 appropriate function nodes are typically selected (25). In this study, three nodes are selected for analysis.

#### 2.3.2 Quantile regression

Quantile regression is a generalized regression analysis method, through the traditional least squares regression. In line with the goal of this study, which is to determine how the influence of explanatory variables changes at various levels of survival risk, this tool is used to estimate the relationship between various quartiles of the response variable (e.g., 25% quartile, 50% quartile, 75% quartile) and various predictor variables. As a result, Therefore, quantile regression not only shows the distribution characteristics of the data, provides a more comprehensive view of how the predictors affect the response variables, and in the actual data collection process, quantile regression can be in the measurement error or extreme cases can still maintain stable estimates, give reliable results. For quantile regression, the minimum weighted absolute deviation is as follows:


$$\min\{w_{t}|y_{t}-\text{α}|\}=-\sum_{i:y_{i}<\alpha }^{T}\left(1-\tau )(y_{t}-\alpha \right)+\sum_{i:y_{i}\geq \alpha }^{T}\tau (y_{t}-\alpha )$$

#### 2.3.3 Bayesian age period cohort model

The Bayesian age-period cohort model is a complex model architecture that combines Bayesian statistical inference methods with age, period, and cohort factors analysis. It is based on the Bayes theorem, which updates the probability distribution judgments of unknown parameters in combination with newly observed data. The future burden of disease for particular ages, periods, and cohorts is predicted using Bayesian age, period, cohort (BAPC) models. The model can forecast patterns in disease incidence or mortality by modeling various future situations. The model’s parameters are estimated using the Markov Chain Monte Carlo (MCMC) method for Bayesian inference, which is based on Leaf Bayesian statistics (26, 27). In addition to being a more robust predictive model due to its ability to overcome the convergence issues associated with Markov Chain Monte Carlo sampling methods in traditional Bayesian methods, the BAPC model is specifically designed to predict cancer incidence and mortality and is better able to predict long-term trends in cancer incidence and mortality, which aids in the planning of future public health resources and interventions.

## 3 Results

### 3.1 Burden of CRC due to smoking, high body mass index, and alcohol use

**Figure 1:**
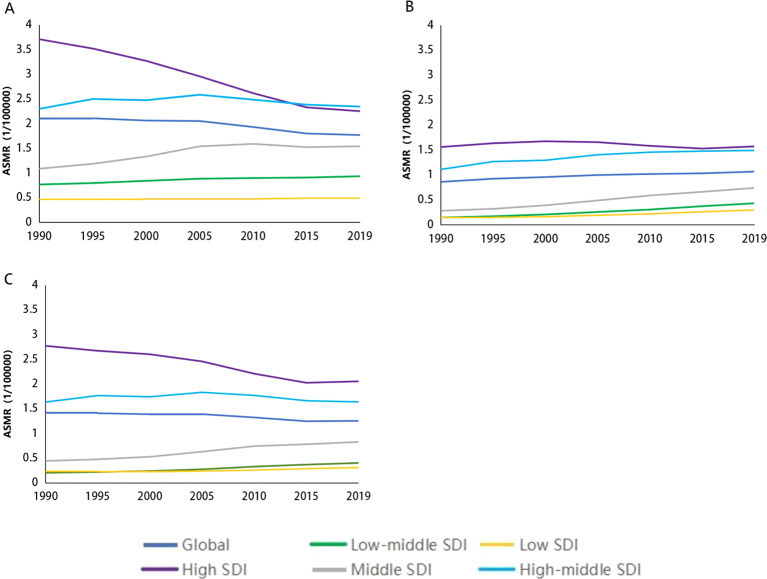
Age-standardized mortality rates of colorectal cancer due to smoking, alcohol use, high body mass index at different SDI levels from 1990 to 2019. **(A)** age-standardized mortality rate of colorectal cancer due to smoking al different SDI levels from 1990 to 2019. **(B)** ASMR of colorectal cancer due to high body mass index at different SDI levels from 1990 to 2019. **(C)** ASMR of colorectal cancer due to alcohol use at different SDI levels from 1990 to 20 19. ASMR 1/4 age-standardized mortality; SDI 1/4 sociodemographic index.

We collected data on smoking-, alcohol-, and obesity-related colorectal cancer mortality rates (ASMRs) and death rates (ASDRs) for five different Social Development Index (SDI) quintiles between 1990 and 2019. **Figure 1A** illustrates the global trends in ASMRs for CRC attributable to smoking. The data indicate that from 1990 to 2019, ASMRs for colorectal cancer decrease by 16.00% (from 2.11 to 1.77 per 100,000 individuals). In the high-SDI quintile population, the mortality rate decreases by 39.27% (from 3.71 per 100,000 to 2.25 per 100,000). **Figure 1B** illustrates changes in ASMRs for obesity-related colorectal cancer attributed to high body mass index. The data indicate that from 1990 to 2019, the ASMRs for colorectal cancer increase by 23.25% (from 0.86 to 1.07 per 100,000). In terms of colorectal cancer-related mortality, the high-SDI quintile population is relatively less affected by high body mass index. The largest increase in ASMRs is found in **Figure 1C** for countries in the middle-SDI, increase from 0.44/100,000 to 0.83/100,000. Conversely, the largest decrease is noted in low-SDI, decreased from 2.77/100,000 to 2.05/100,000 (**Figure 1C**).

**Figure 2:**
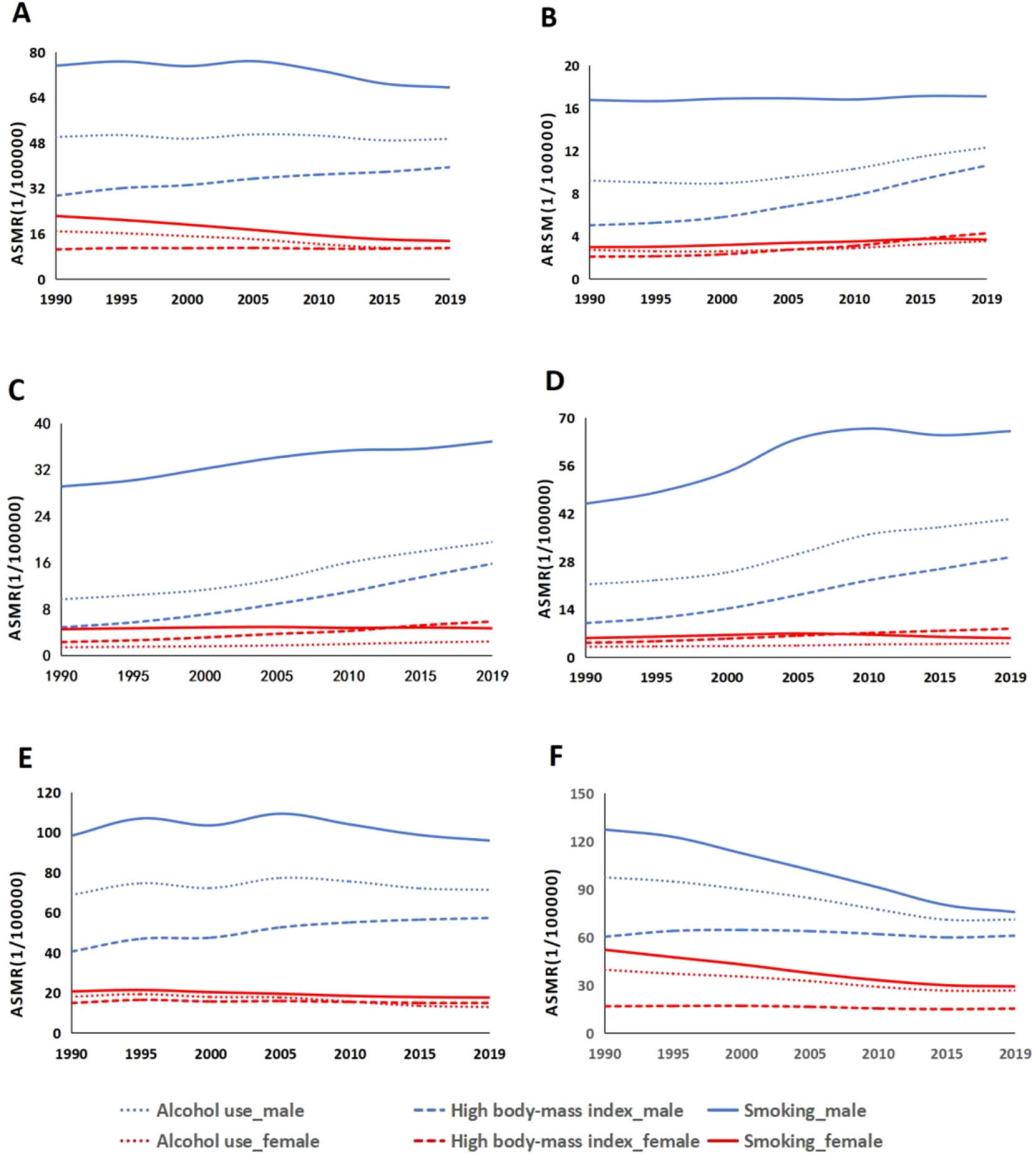
Age-standardized mortality rates for colorectal cancer due to smoking, high body mass index, and alcohol use at different sociodemographic index (SDI) levels, 1990-2019: **(A)** global, **(B)** low SDI, **(C)** low-to-moderate SDI, **(D)** moderate SDI, **(E)** high-to-moderate SDI, and **(F)** high SDI.ASMR 1/4 age-standardized mortality rates.

This study found that globally men have higher ASMRs for colorectal cancer than women. In 2019, the ASMRs attributable to smoking were 67.49 per 100,000 men and 13.40 per 100,000 women. The ASMRs due to alcohol use were 49.40 per 100,000 men and 10.74 per 100,000 women. As for high body mass index, the ASMRs were 39.40 per 100,000 men and 10.97 per 100,000 women (**Figure 2A**). Between 1990 and 2019, across various levels of the SDI, the primary contributors to ASMRs for CRC, listed from the highest to the lowest, are smoking, alcohol consumption, and having a high body mass index. The ASMRs associated with alcohol consumption, smoking, and high body mass index in men showed increasing trends in low, low-middle, and middle SDI (**Figures 2B–D**), which is comparable to the pattern seen in middle-high SDI quintile countries. The ASMRs for colorectal cancer linked to smoking, alcohol consumption, and having a high body mass index show a downward trend in both males and females in high SDI quintile nations (**Figure 2E**). In 2019, the medium-high SDI region had the most ASMR for smoking-related colorectal cancer in men (95.89/100,000), while the high SDI region had the highest ASMR for women (29.16/100,000)(**Figure 2F**). In 2019, communities with medium-high SDI had the highest ASMR for alcohol-attributable colorectal cancer in men (71.32/100,000), while the high SDI group had the highest ASMR for women (26.63/100,000). In 2019, the High SDI group had the highest ASMR for colorectal cancer related to high body mass index among women (15.32/100,000), whereas the High SDI group had the highest ASMR for males (60.84/100,000). Men’s and women’s ASDR of colorectal cancer displayed traits comparable to ASMR in five distinct SDI quartiles (**Figure 2**).

**Figure 3:**
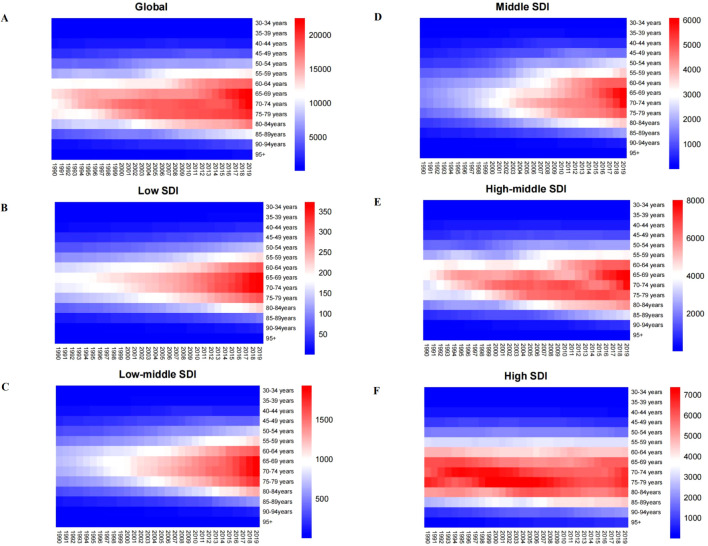
Age-specific mortality rates (per 100,000 people) of colorectal cancer attributable to smoking at different SDI levels from 1990 to 2019.SDI ¼ sociodeinographic index. **(A)** global; **(B)** low SDI; **(C)** low to middle SDI.

We collected data from five different SDI regions around the world and analyzed age-specific mortality rates from colorectal cancer due to tobacco use, alcohol use, and high body mass index, and heat maps depicting these mortality rates are shown in **Supplementary Figures S1, S2**; **Figure 3**. The chart analysis indicates that, across all SDI regions, the age group with the greatest age-specific mortality rate for smoking-attributable colorectal cancer is mainly concentrated between the ages of 70 and 74 (**Figure 3A**). ASMRs for CRC caused by smoking are often greater than those caused by alcohol intake and high body mass index on a global scale (**Supplementary Figures S1A, S2A**). In 2019, the ASMRs for individuals aged 70 to 74 was 14,086.80 per 100,000 population (**Figure 3A**). Among the top five regions, colorectal cancer caused by alcohol abuse factors and high body mass index tends to show a clear trend of increasing age (**Supplementary Figures S1B-D, S2B-D**). Additionally, regions with higher SDI indices exhibit higher ASMRs for colorectal cancer (**Figures 3B, C**). In low-SDI areas, the age-specific mortality rate for this cancer is the lowest, with a rate of 200.36 per 100,000 people aged 70 to 74 in 2019 (**Figure 3D**). In high-SDI regions, the ASMRs for those aged 70 to 74 in 2019 was 5,548.32 per 100,000 population (**Figures 3E, F**). The ASMRs in high and high-middle SDI regions are similar, while those in low SDI regions are significantly lower (**Supplementary Figures S1E-F**).

### 3.2 Between SDI and ASMR and ASDR of CRC

**Figure 4:**
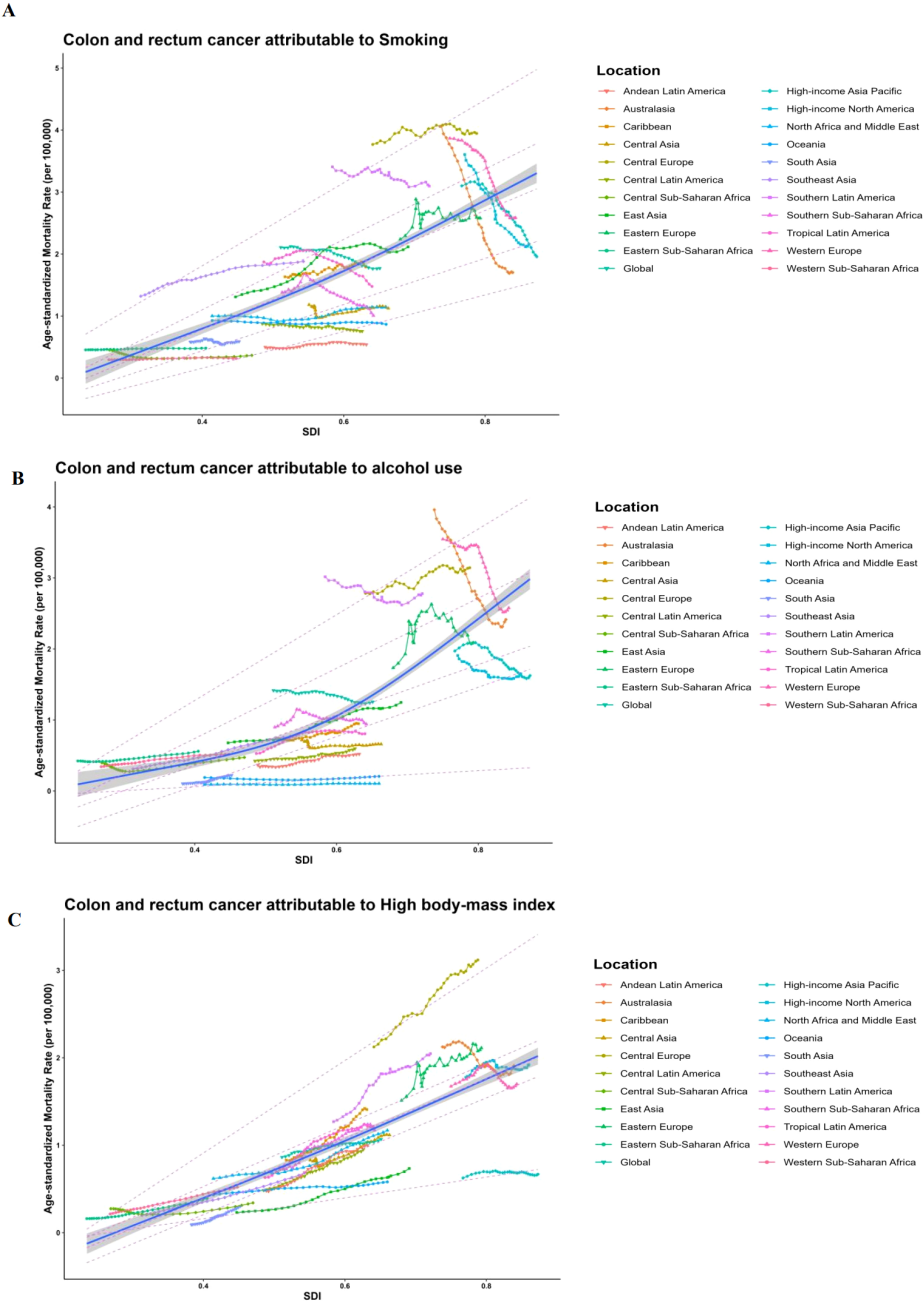
Long-term associations (1990-2019) of age-standardized mortality rates (per 100,000 population) for colorectal cancer with smoking, alcohol use, high body mass index, and SDI for 21 regions worldwide, classified by global burden of disease. Each colored line represents the time trend for the given region, and each dot represents a specific year for that region. **(A)** Long-term associations (1990-2019) of ASMRs (per 100.000 population) for CRC with smoking; **(B)** Long-term associations (1990-2019) of ASMRs (per 100,000 population) for CRC with alcohol use; **(C)** Long-term associations (1990-2019) of ASMRs (per 100,000 population) for CRC with high body mass index; Dashed lines represent the results of quantile regressions; from top to bottom, the results are for P95, P75, P50, P25, and P5. p5 1/4 5th percentile; p25 1/4 25th percentile; p50 1/4 50th percentile; p75 1/4 75th percentile; p95 1/4 95th percentile; and sdi 1/4 sociodemographic index.

ASMR data and SDI values are gathered from 21 regions based on the GBD categorization. RCS and quantile regression analysis are used to investigate the connection between ASMR and SDI. The RCS findings when the SDI is around 0.8 are shown in **Figure 4**. The medium SDI quintile level is where the largest ASMR for colorectal cancer attributable to smoking variables is seen (**Figure 4A**). High body mass index is linked to reduced ASMR for colorectal cancer with rising SDI, however alcohol usage is linked to increased ASMR (**Figures 4B, C**). The relationship between ASDR and SDI yields similar results. The five quantiles are statistically significant. The ASMR of colorectal cancer attributed to smoking, alcohol use, and high BMI increases with rising SDI (**Figure 4**). The link between ASDR and SDI yields similar findings.

### 3.3 ASMR forecast for CRC in different SDI countries, 2019 - 2030

The Bayesian age-period-cohort model was used to predict trends in mortality rates between 2020 and 2030; ASMR data on colorectal cancer attributable to smoking, alcohol use, and high body mass index under different SDI levels (high SDI: USA; High SDI; Middle SDI: China; and Low SDI: Tanzania), including China, India, and the United States, Italy and Tanzania, representing different SDI levels. It provides better coverage and accuracy compared to other prediction methods.

**Figure 5:**
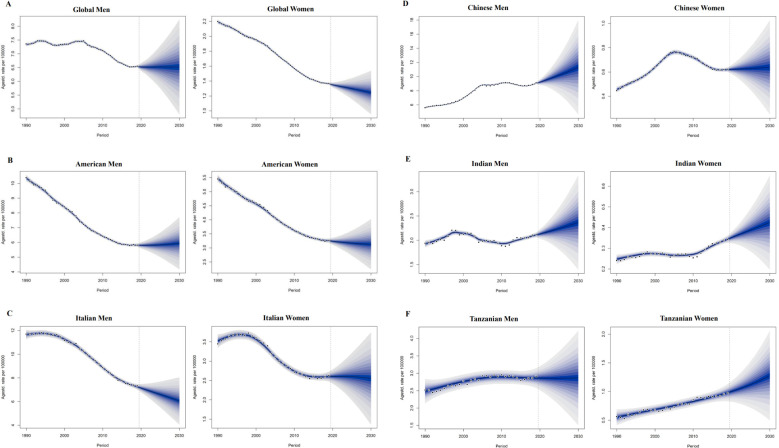
5.1 Projections of ASDR of colorectal cancer attributable to smoking in different sociodemographic index countries from 2020 to 2030. ASDR 1/4 age-standardized DALYs rate. **(A)** global men and women; **(B)** American men and women; **(C)** Italian men and women; 5.2 Projections of ASDR of colorectal cancer attributable to smoking in different sociodemographicindex countries from 2020 to 2030. ASDR 1/4 age-standardized DALYs rate. **(A)** global men and women; **(B)** American men and women; **(C)** Italian men and women; **(D)** Chinese men and women; **(E)** Indian men and women; **(F)** Tanzanian men and women.

The projected trends in ASMR for colorectal cancer attributable to smoking, alcohol use and high body mass index over the next decade in countries of different SDI levels are shown in **Figure 5**, **Supplementary Figures S3, S4**. In most regions, the ASMR for colorectal cancer due to three risk factors shows a decreasing trend. However, the ASMR for colorectal cancer caused by smoking is significantly higher than that caused by alcohol use across countries with varying SDI levels (**Supplementary Figure S3A**). In 2019, the ASMR for CRC attributed to smoking are higher than the global average in regions with SDI levels of the United States and Italy; In contrast, the ASMR for CRC in regions with SDI levels of China, India, and Tanzania are lower than the global average (**Figure 5**).

The global age-standardized mortality rate (ASMR) for colorectal cancer linked to alcohol use in men fell by 11.27% between 1990 and 2019, from 1.42 (95% CI:1.09~1.74) in 1990 to 1.26 (95% CI:0.95~1.58) in 2019. From 2020 to 2030, the global ASMR for colorectal cancer linked to alcohol use in men is expected to rise by 8.88% (95% CI: -18.86% to 35.05%) (**Table 1**). In high SDI regions, the ASMR for CRC decreases by 25.99% (95% CI: 25.35% to 25.59%). The ASMR for CRC attributed to alcohol consumption in women increases by 1.43% (95% CI: -29.41% to 30.56%). In the prediction of colorectal cancer due to high body mass index.Among the five different SDI quintiles, the ASMR for CRC remains relatively stable in regions with high,high-middle SDI (**Supplementary Figures S4B, S4C**). On the other hand, ASMR rises in low-middle, medium, and SDI locations, with the biggest increase shown in low-middle SDI regions (**Supplementary Figures S4D-F**). In 2019, the age-standardized mortality rate (ASMR) for colorectal cancer in high and high-middle SDI regions is higher than the global average (**Supplementary Figures S3B, S3C**). Conversely, the ASMR in medium, low-middle, and low SDI regions is lower than the global average (**Supplementary Figure S3D**). But in Tanzania, China, India, and the United States, the ASMR for CRC linked to alcohol use rises for both men and women. Among these countries, India exhibits the highest increase in ASMR for both men and women (**Supplementary Figure S3E**). The ASMR from alcohol consumption in Italian males and females shows a decreasing trend in colorectal cancer (**Supplementary Figure S3F**). The ASMR for smoking-attributable colorectal cancer in men is expected to decline by -0.03% (95% CI: -29.89% to 23.57%) in comparison to worldwide predictions for 2020 to 2030 (**Figure 5A**); In females, the ASMR for colorectal cancer attributable to smoking is also projected to decrease, showing a percentage change of -7.41% (95% CI: -92.42% to 10.87%). The ASMR forsmoking-related colorectal cancer in men is declining in Italy (**Figure 5B**). On the other hand, the ASMR for smoking-related colorectal cancer in men rises in Tanzania, India, China, and the United States, with China showing the most increase (**Figure 5C**). In women, the ASMR for smoking-attributable colorectal cancer declines in the US and Italy, with the US showing the biggest fall. On the other hand, China, India, and Tanzania all saw increases in the ASMR for colorectal cancer linked to smoking in women, with Tanzania seeing the largest rise (**Figures 5E, F**).

**Table 1:** Disease burden of colorectal cancer attributable to smoking, alcohol abuse, and obesity in 1990-2019.

SDI region	Deaths Cases (95% UI)	ASMR (1/100000)	DALYs (95%UI)	ASDR (1/100000)
Alcohol use-1990				
Global	33239 (25537-41330)	1.42 (1.09-1.74)	1304642 (1003803-1598615)	32.17 (24.76-39.47)
Low SDI	305 (181-421)	0.23 (0.17-0.31)	16264 (11666-21619)	3.08 (2.21-4.09)
Low-middle SDI	531 (371-700)	0.20 (0.15-0.27)	39730 (29508-51649)	3.52 (2.61-4.57)
Middle SDI	10984 (8487-13571)	0.44 (0.34-0.56)	144153 (108785-183544)	8.4 (6.34-10.69)
High-middle SDI	3859 (2841-4907)	1.64 (1.26-2.02)	445192 (343218-549320)	38.7 (29.83-47.75)
High SDI	17506 (13039-22012)	2.77 (2.13-3.4)	658694 (509035-804387)	80.13 (61.92-97.86)
Alcohol use-2019				
Global	52495 (40436-64250)	1.26 (0.95-1.58)	2406874 (1830856-3002940)	29.07 (22.09-36.24)
Low SDI	550 ( 397-729)	0.31 (0.22-0.41)	46392 (33077-61381)	7.88 (5.62-10.35)
Low-middle SDI	1268 ( 943-1655)	0.4 (0.3- 0.52)	159999 (118303-207088)	10.77 (7.96-13.96)
Middle SDI	4647 (3500-5926)	0.83 (0.6-1.09)	571344 (418805-752801)	21.64 (15.83-28.49)
High-middle SDI	17073 (13152- 21154)	1.64 (1.23-2.08)	806144 (603326-1022211)	39.92 (29.86-50.58)
High SDI	28932 ( 22222-35468)	2.05 (1.59- 2.53)	821897 (640097-1015353)	47.60 (37.18-58.91)
High body-mass index-1990				
Global	31896 (3511-15666)	0.86 (0.42-1.45)	769648 (382060-1284465)	19.14 (9.49-31.99)
Low SDI	329 (108-705)	0.15 (0.05- 0.32)	9401 (3146-19955)	3.6 (1.19-7.68)
Low-middle SDI	842 (285-1787)	0.15 (0.05- 0.32)	24018 (8284-50676)	3.65 (1.24-7.74)
Middle SDI	2795 (1112-5534)	0.28 (0.11- 0.56)	80746 (32320-159857)	7.08 (2.83-13.99)
High-middle SDI	11557 (5944-18768)	1.11 (0.57-1.82)	287385 (148698-465637)	26.18 (13.51-42.52)
High SDI	16354 (8198-26809)	1.56 (0.78-2.56)	367682 (186195-593603)	36.05 (18.32-58.1)
High body-mass index-2019				
Global	85882 (46847-136520)	1.07 (0.58-1.7)	2021536 (1121762-3184394)	24.41 (13.52-38.55)
Low SDI	1505 (727-2605)	0.31 (0.22-0.41)	42868 (20976-73730)	7.41 (3.61-12.78)
Low-middle SDI	5761 (2894-9631)	0.4 (0.3-0.52)	155685 (79127-259661)	10.72 (5.43-17.89)
Middle SDI	18014 (9044-30081)	0.83 (0.6-1.09)	483737 (247153-801985)	18.46 (9.39-30.68)
High-middle SDI	30303 (16707-47262)	1.64 (1.23-2.08)	700897 (392052-1089989)	34.51 (19.26-53.73)
High SDI	30247 (16530-46903)	2.05 (1.59-2.53)	637133 (358980-965326)	36.99 (21.04-55.77)
Smoking-1990				
Global	78784 (52851-104663)	2.11 (1.39-2.83)	1856999 (1226908-2432756)	46.45 (30.9-61.03)
Low SDI	957 (447-1482)	0.47 (0.23-0.71)	23444 (9810-37019)	9.98 (4.56-15.49)
Low-middle SDI	4109 (2320-5789)	0.77 (0.45-1.08)	103187 (54717-148683)	16.96 (9.36-24.09)
Middle SDI	10253 (6236-13743)	1.09 (0.69-1.45)	261270 (151049-354670)	24.6 (14.51-33.14)
High-middle SDI	24234 (16274-31905)	2.3 (1.54-3.05)	596030 (380535-783088)	54.23 (34.98-71.30)
High SDI	39190 (24996-53396)	3.71 (2.37-5.03)	872175 (579708-1165668)	84.91 (56.87-112.6)
Smoking-2019				
Global	142931 (95516-193475)	1.77 (1.19-2.4)	3226829 (2088871-4402205)	38.87 (25.33-52.98)
Low SDI	2211 (1032-3252)	0.49 (0.23-0.72)	52151 (22489-78132)	10.27 (4.66-15.17)
Low-middle SDI	11889 (7130-16455)	0.94 (0.56-1.29)	277597 (157660-390009)	20.14 (11.57-28.04)
Middle SDI	36865 (23768-50683)	1.54 (1.01-2.11)	887887 (539525-1244023)	34.50 (21.5-48.11)
High-middle SDI	48208 (31968-65104)	2.35 (1.56-3.17)	1101633 (706214-1493802)	53.39 (34.23-72.42)
High SDI	43684 (26787-61606)	2.25 (1.42-3.14)	905929 (586806-1243664)	51.07 (33.47-69.22)

## 4 Discussion

In the current study on the burden of colorectal cancer, qualitative and quantitative methods, including systematic reviews and meta-analyses, Global Burden of Disease data, and Joinpoint models, are employed to perform an in-depth analysis of the disease burden attributable to risk factors leading to cancer (28–30). However, Long-term data trends are provided by these studies, facilitating the understanding of the evolution of the colorectal cancer burden over time and its variations across different regions and countries. This study aims to fill gaps in current research by conducting an in-depth analysis of specific colorectal cancer risk factors, including smoking, alcohol consumption, and obesity, across 21 regions and five social development index (SDI) levels. Insights into the impact of these risk factors on the disease burden in different regions are provided. Our research indicates that from 1990 to 2019, the ASMR and the ASDR due to smoking and heavy alcohol consumption show a declining trend globally. In regions with a high SDI, this trend remains consistent; however, in medium, low-medium, and low SDI regions, an increasing trend is observed. Additionally, the ASMR and ASDR due to smoking are significantly higher compared to the corresponding rates attributed to alcohol consumption and high body mass. Data analysis indicates that, consistent with our research findings, the ASMR and ASDR of CRC caused by smoking show an upward trend in most countries globally, particularly in developing countries. Additionally, a negative correlation is observed between the SDI and changes in ASMR and ASDR (31, 32). This disparity is primarily attributed to the effective implementation of smoking cessation policies and the widespread availability of medical screening and early diagnosis in high SDI regions, thereby reducing the health burden in these areas (33). To address this disparity, global collaboration and partnerships are crucial in improving healthcare infrastructure and enhancing health awareness in underdeveloped regions. Similarly, from 1990 to 2019, the ASMR and the ASDR due to obesity show an upward trend globally and across different levels of SDI. A substantial body of both prospective and retrospective research demonstrates significant association between overweight and obesity and the incidence and mortality of colorectal cancer (34). The promotion of cancer screening, early detection and treatment, unhealthy lifestyles, the effects of economic and social development, and the rise in obesity rates worldwide are the main causes of this trend. Globally, public health policymaking and implementation must be improved to counter these trends, particularly in areas with low and medium-to-poor SDIs. The “Global Action Plan for the Prevention and Control of Noncommunicable Diseases 2013-2020” was released concurrently by the World Health Organization (WHO) to address this problem. The plan aims to reduce premature mortality from noncommunicable diseases by targeting various risk factors, including smoking, alcohol consumption, and unhealthy diets (35). The significance of mitigating the cancer burden through health promotion and disease prevention strategies is emphasized by the plan.

Current studies indicate that smoking, alcohol consumption, and obesity are the primary modifiable risk factors closely associated with CRC. However, the research on gender and age differences in the impact of these three risk factors on CRC progression remains relatively limited (36, 37). Our study demonstrates that in regions with varying SDI levels, the ASMR of CRC due to alcohol consumption and smoking is significantly higher in males than females. The differences between genders remain significant, especially in high SDI regions, with the disparity due to smoking being more pronounced. The gender disparity is determined by physiological differences, socio-cultural factors, and environmental influences. Additionally, it is observed that the ASMR of CRC attributable to obesity have shown an upward trend among males across different Socio-Demographic Index (SDI) regions from 1990 to 2019. According to WHO data, one of the main causes of the rise in ASMR and ASDR of colorectal cancer associated with obesity is the global increase in obesity rates. This phenomenon is closely related to unhealthy lifestyles and dietary habits, which are becoming increasingly common worldwide (38, 39). Research indicates that, from 1990 to 2019, the ASMR for CRC in individuals aged 65 to 74 is at its highest due to alcohol consumption, smoking, and obesity. This trend demonstrates a continuous increase in regions with medium-to-high and high SDI levels. Older adults typically have longer exposure periods to risk factors such as smoking and alcohol consumption. As a result, with increasing age, the health impacts of these behaviors accumulate, thereby elevating the risk of CRC. It is noteworthy that since the 1990s, the mortality rate of colorectal cancer in individuals under 50, attributable to risk factors such as alcohol consumption, smoking, and obesity, has shown a gradual upward trend globally. It is noteworthy that since the 1990s, the mortality rate of CRC in individuals under 50, attributable to risk factors such as alcohol consumption, smoking, and obesity, has shown a gradual upward trend globally (40, 41), Additionally, obesity and physical inactivity are considered the primary causes of CRC in younger adults.

In exploring the relationship between ASMR and SDI across 21 regions, smoking is identified as the highest contributing factor to CRC mortality. Among the risk factors of smoking, alcohol consumption, and obesity, Eastern Europe exhibits the highest mortality rate, while South Asia shows the lowest (41–43). According to our research, smoking and CRC mortality are significantly correlated. As the SDI increases, the global cancer burden attributed to smoking also rises. In nations going through significant changes, the death rate of colorectal cancer frequently corresponds with the Human advancement Index (HDI), making it a key measure of socioeconomic advancement. In Eastern Europe, food and lifestyle changes may raise the risk of CRC. In contrast, insufficient medical resources and limited access to treatment in South Asia may result in inadequate diagnosis and treatment of colorectal cancer, with some cases remaining unrecorded. Consequently, the mortality rate from colorectal cancer in this region appears relatively low. Additionally, it is observed that in various socio-development indices, the mortality rate from colorectal cancer attributable to obesity shows an increasing trend across 21 regions. This phenomenon is influenced by the global rise in obesity rates, unhealthy lifestyles, and the policies and guidelines of the WHO. Cancer screening and early diagnosis and treatment are also promoted. Furthermore, social and economic development are important. Globally, public health strategies must be developed and implemented more effectively to counteract this tendency, especially in areas with medium and low SDI (44, 45). According to the forecasts, the burden of smoking-related colorectal cancer mortality is expected to decrease among both men and women worldwide, as well as in the US and Italy, over the course of the next ten years. The global burden of male colorectal cancer mortality has started to decline, especially after 2008. This shift is directly related to the adoption of the MPOWER comprehensive strategy, which aims to reduce tobacco use and was described in the WHO’s 2008 Global Tobacco Epidemic Report (46). Previous studies indicate that since 1960, proactive measures have been implemented to promote CRC screening in the US. By 2021, the overall screening coverage for individuals aged 50 and above has reached 70% (47). Furthermore, it is anticipated that China, India, and Tanzania will see an increase in the burden of colorectal cancer mortality linked to drinking and smoking. The trend is thought to be directly linked to inadequate policies for alcohol and tobacco control, as well as a lack of efficient colon cancer screening and early diagnosis procedures. Therefore, lowering the burden of colorectal cancer mortality in these areas requires the adoption of efficient alcohol control measures, the encouragement of healthy lifestyles, and the improvement of CRC screening and early detection. Globally, the majority of disability-adjusted life years (DALYs) from colorectal cancer originate from years of life lost (YLL). Consequently, the trends in ASMR and ASDR are consistent. On the other hand, the research results have pioneering practical significance, based on Bayesian age cohort modeling to accurately predict the future burden of colorectal cancer disease attributable to risk factors of tobacco use, alcohol abuse, and high body mass index. A dual-track approach of tobacco control and healthy lifestyle promotion was proposed for regions with high smoking prevalence and obesity. These strategies are expected to help the plight of colorectal cancer prevention and control in different regions.

The accuracy of colorectal cancer burden models is closely linked to the quality of global disease burden data. The variations in data collection methods throughout various nations and areas are likely to impact the comparability of data. Furthermore, specific parameters are sometimes omitted in query results, resulting in data incompleteness. GBD 2019 reveals that key risk factors such as smoking, alcohol consumption, and obesity are increasing globally, contributing significantly to the increase in-communicable diseases. However, inconsistencies are observed in the effectiveness of public health policies aimed at addressing and controlling these risk factors (20). Given that some low- and middle-income nations do not have efficient health monitoring systems, this study was designed to forecast future illness loads. This shortcoming affects the data’s dependability and quality, which reduces the precision of predictive analysis.

## 5 Conclusions

In the end, colorectal cancer (CRC) is still a serious public health concern that needs immediate worldwide attention and consideration. Our research demonstrates that although the ASDR from CRC caused by smoking and drinking is decreasing, the ASDR due to these factors significantly increases in low, lower-middle, and middle SDI regions. Furthermore, the prevalence of colorectal cancer linked to obesity is steadily increasing. Furthermore, men bear a disproportionately high burden of smoking-related colorectal cancer mortality, and this gender gap widens over time. Notably, an increasing trend in the burden of colorectal cancer due to smoking and alcohol consumption is observed in younger age groups. This study fully examines the patterns of colorectal cancer related to alcohol and cigarettes usage and obesity over the previous three decades. The findings emphasize the importance of preventing colorectal cancer through the control of modifiable risk factors and highlight the necessity of developing targeted public health strategies in regions with differing SDI levels. The study simultaneously lays a foundation for future research in this field, anticipating advancements in the prevention, early diagnosis, and treatment of colorectal cancer.

## Data Availability

Publicly available datasets were analyzed in this study. This data can be found here: https://vizhub.healthdata.org/gbd-results/.
