# Supplementary material for: Global, Regional, and National Disease Burden and Prediction Analysis of Colorectal Cancer Attributable to Tobacco, Alcohol, and Obesity From 1990 to 2030
Source: Front Oncol. 2025 Feb 26;15:1524308. doi: 10.3389/fonc.2025.1524308 (PMC11896864; doi:10.3389/fonc.2025.1524308)
Supplement: Supplementary file 1 [file DataSheet1.docx]

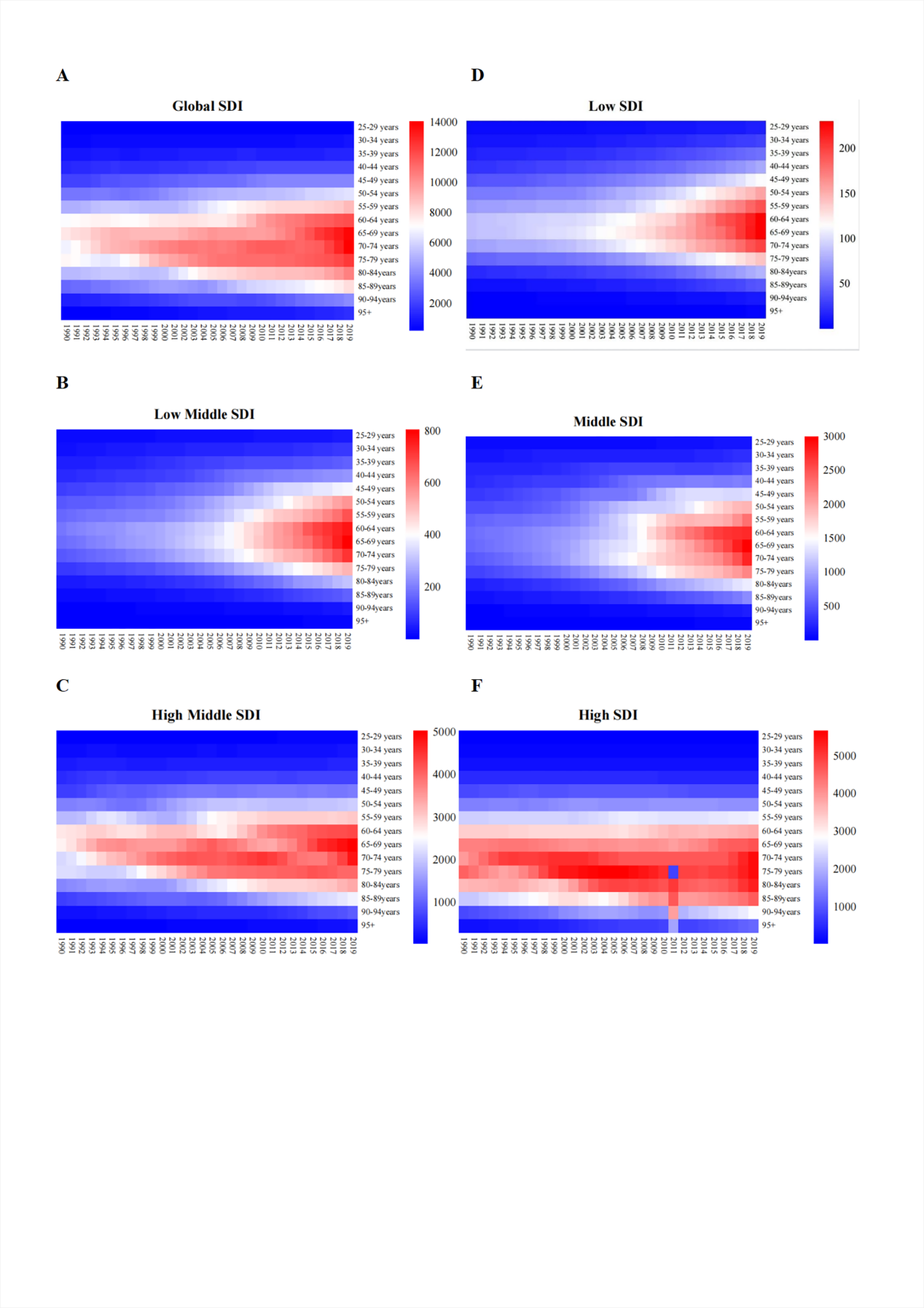


Figure S1 Age-specific mortality (per 100,000 people) of colorectal cancer attributable to Alcohol use at different SDI levels from 1990 to 2019. SDI ¼ sociodemographic index. (A) global, (B) low SDI, (C) low-to-moderate SDI, (D) moderate SDI, (E) high-to-moderate SDI, and (F) high SDI.


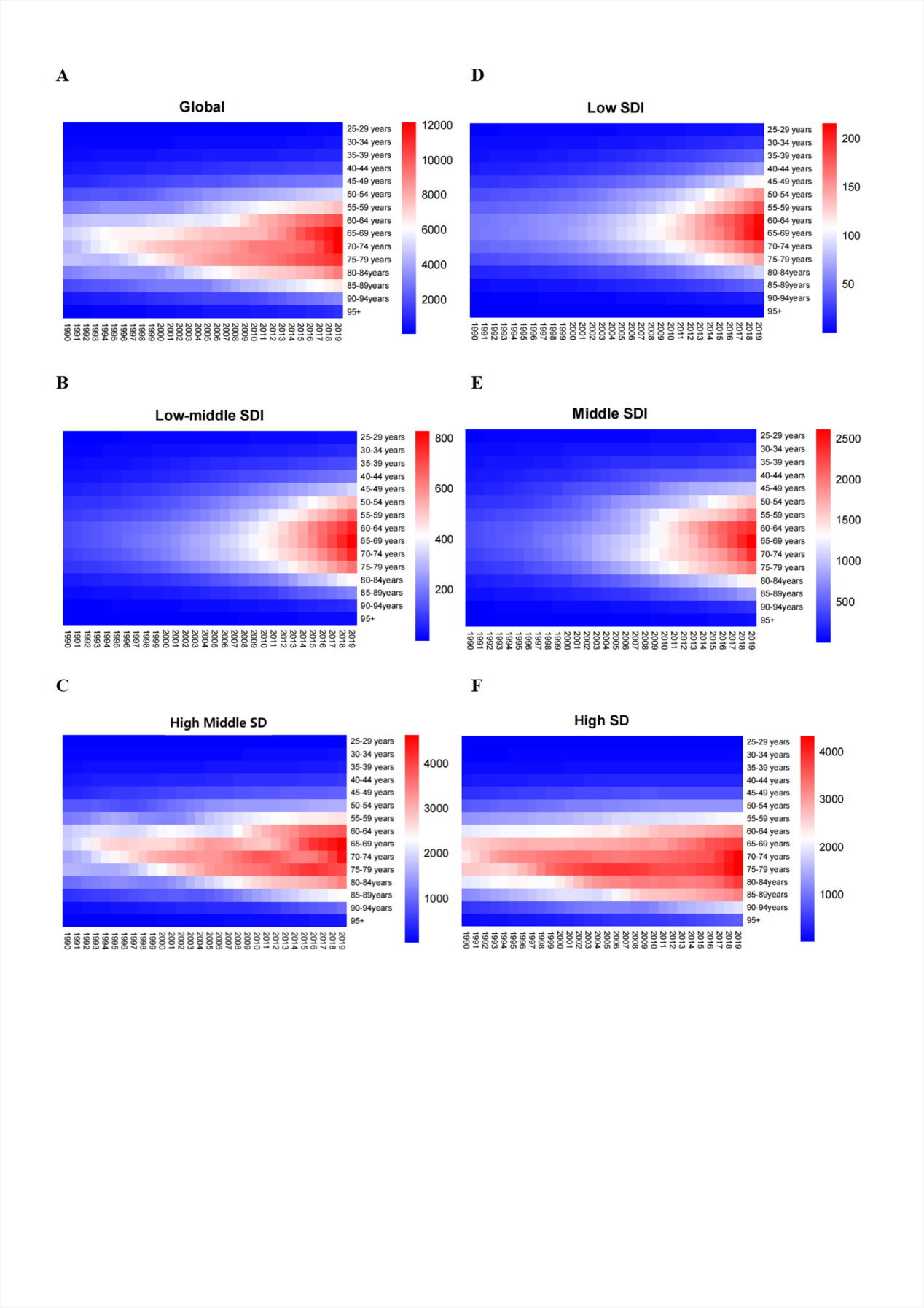


Figure S2 Age-specific mortality (per 100,000 people) of colorectal cancer attributable to High body-mass index at different SDI levels from 1990 to 2019. SDI ¼ sociodemographic index. (A) global, (B) low SDI, (C) low-to-moderate SDI, (D) moderate SDI, (E) high-to-moderate SDI, and (F) high SDI.


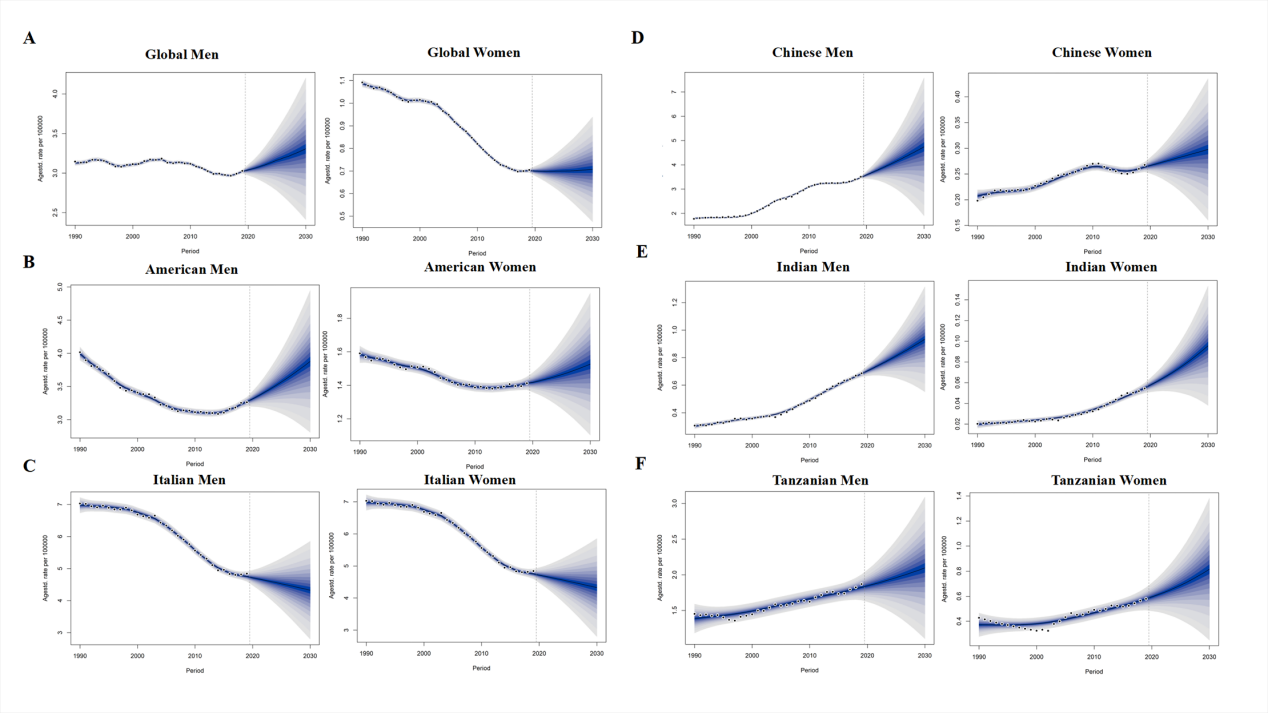


Figure S3 Projections of ASMR of colorectal cancer attributable to alcohol use in different sociodemographic index countries from 2020 to 2030. ASMR ¼ age-standardized mortality rate. (A) global men and women; (B) American men and women; (D) Chinese men and women; (E) Indian men and women; (F) Tanzanian men and women.


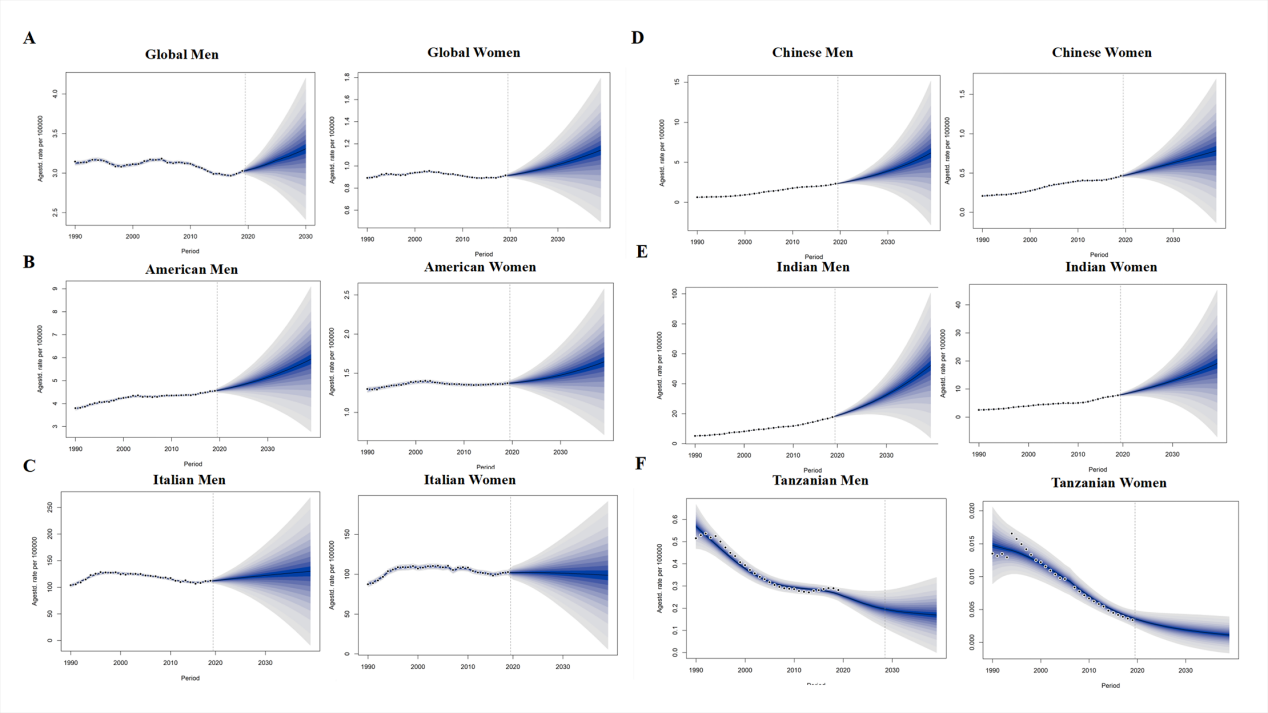


Figure S4 Projections of ASMR of colorectal cancer attributable to high body mass index in different sociodemographic index countries from 2020 to 2030. ASMR ¼ age-standardized mortality rate. (A) global men and women; (B) American men and women; (D) Chinese men and women; (E) Indian men and women; (F) Tanzanian men and women.
